# Neodymium-Doped Yttrium Aluminum Garnet (Nd:YAG) Laser-Induced Damage to Intraocular Lenses: A Systematic Review of Mechanisms, Clinical Consequences, and Preventive Strategies

**DOI:** 10.7759/cureus.90903

**Published:** 2025-08-24

**Authors:** Andreas F Borkenstein, Eva-Maria Borkenstein

**Affiliations:** 1 Ophthalmology, Borkenstein and Borkenstein Private Practice, Privatklinik der Kreuzschwestern Graz, Graz, AUT

**Keywords:** capsulotomy complications, hydrophilic acrylic, hydrophobic acrylic, intraocular lens, iol damage, laser safety, nd:yag laser, posterior capsular opacification, small-aperture iol

## Abstract

Neodymium-doped:yttrium-aluminum-garnet (Nd:YAG) laser capsulotomy is considered the gold standard treatment for visually significant posterior capsule opacification (PCO), which remains the most common long-term complication following cataract surgery. However, Nd:YAG laser treatment carries the risk of inducing intraocular lens (IOL) damage, including pits, cracks, and optical degradation, particularly affecting the quality of premium hydrophobic acrylic and small-aperture IOLs. To systematically review the morphology, material dependence, optical, and clinical consequences, as well as preventive strategies associated with Nd:YAG laser-induced IOL damage. A comprehensive literature search was conducted in PubMed and Medical Literature Analysis and Retrieval System Online (MEDLINE) databases, including publications from January 1995 to April 2025. Keywords and MeSH terms included "Nd:YAG capsulotomy," "intraocular lens damage," "IOL pits," "posterior capsule opacification," "device-related injuries," and "Yttrium Aluminum Garnet." Peer-reviewed experimental studies, clinical series, case reports, reviews, and guidelines published in English or German were included. Non-peer-reviewed articles, editorials, and conference abstracts were excluded. A total of 53 relevant publications were included following Preferred Reporting Items for Systematic Reviews and Meta-Analyses (PRISMA) guidelines. Morphological alterations ranged from superficial micro-pits to extensive crater formation and delamination, particularly prominent in hydrophobic acrylic and pinhole (small-aperture) IOLs. Experimental studies consistently demonstrated measurable degradation in optical quality, including reduced contrast sensitivity, increased glare, and higher-order aberrations after central pit formation. Clinically, symptomatic IOL damage requiring lens explantation was identified in up to 9.3% of cases analyzed retrospectively. Laboratory analyses, including micro-computed tomography (µCT) and Raman spectroscopy, revealed structural and chemical alterations within damaged areas. Preventive measures proven effective include peripheral laser patterns, posterior offset focusing, minimal effective energy usage (<1.8 mJ), and structured laser alignment techniques. Nd:YAG laser-induced IOL damage significantly depends on material properties, lens design, and laser parameters. Effective preventive strategies, including optimized laser protocols and targeted surgical training, can substantially mitigate these risks. However, robust clinical data linking experimental findings to patient outcomes remain scarce, highlighting the need for large prospective studies in the future, especially focusing on modern premium IOLs. Careful attention seems essential to minimize IOL damage, particularly in premium lens designs, thereby ensuring optimal visual outcomes and patient satisfaction.

## Introduction and background

Posterior capsule opacification (PCO), often referred to as "secondary cataract," remains the most common long-term complication following extracapsular cataract extraction, including modern cataract surgeries like phacoemulsification. Its incidence ranges from 20% to 40% within two to five years, depending on factors such as patient age, intraocular lens (IOL) material and design, and surgical technique [1,2]. PCO develops due to the proliferation and migration of residual lens epithelial cells (LECs) onto the posterior capsule, eventually leading to opacification of the visual axis. Neodymium-doped:yttrium-aluminum-garnet (Nd:YAG) laser capsulotomy is considered the gold standard treatment for visually significant PCO. It is typically performed 18 to 36 months postoperatively [3]. A recent population-based study showed that 33.0% of patients undergo Nd:YAG capsulotomy within two years of cataract surgery, and nearly 10% within the first year [4]. Given its widespread use, with an estimated 1.8 million procedures annually across Europe and North America, Nd:YAG capsulotomy represents one of the most frequently performed ophthalmic interventions worldwide [5].

While generally safe and effective, Nd:YAG laser treatment is not without risk. Potential complications include IOL pitting, decentration, pigment dispersion, intraocular pressure (IOP) spikes, and even IOL subluxation [6-9]. These risks become particularly relevant in patients with so-called “premium IOLs”, such as multifocal, toric, or small-aperture optics, which are more susceptible to visual degradation due to their complex optical designs [10, 11]. Misplaced or excessive laser energy can result in optical breakdown and shockwave propagation within the IOL, causing permanent damage [12-14]. As the implantation of premium IOLs continues to rise, understanding and preventing Nd:YAG-associated IOL damage has gained increasing clinical importance [15,16].

This review synthesizes published evidence on Nd:YAG-induced IOL damage across materials and optic designs, with emphasis on morphological effects, clinical impact, and prevention.

Methods

**Figure 1:**
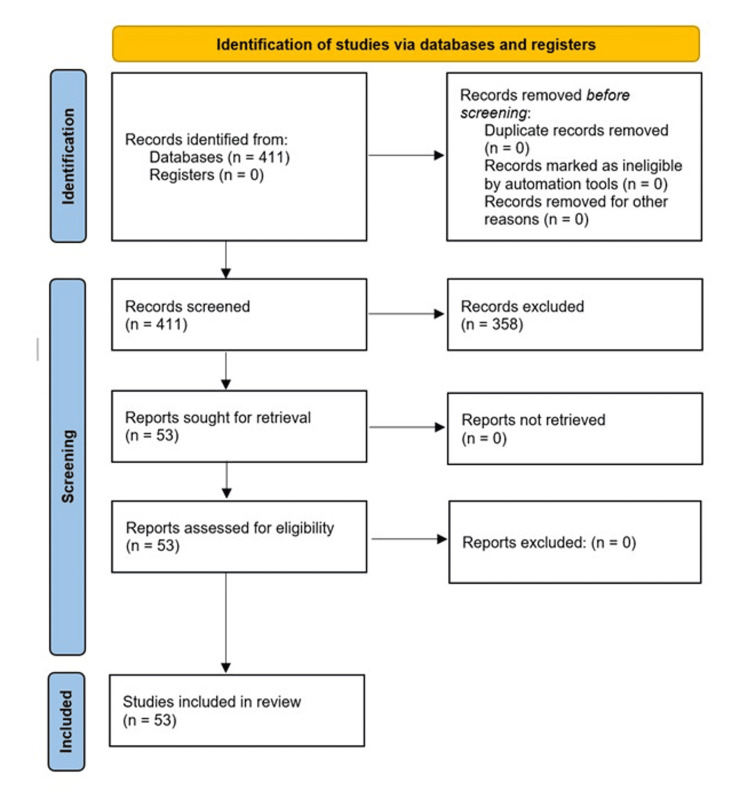
A PRISMA 2020 flow diagram illustrating the identification, screening, and inclusion process of studies on Nd:YAG laser-induced intraocular lens damage PRISMA: Preferred Reporting Items for Systematic Reviews and Meta-Analyses; Nd:YAG: neodymium-doped:yttrium-aluminum-garnet

This systematic review was performed in accordance with Preferred Reporting Items for Systematic Reviews and Meta-Analyses (PRISMA) guidelines. PubMed and Medical Literature Analysis and Retrieval System Online (MEDLINE) databases were systematically searched from January 1995 to April 2025 using the following search strategy: ("Nd:YAG capsulotomy" OR "YAG laser") AND ("intraocular lens damage" OR "IOL pit" OR "posterior capsule opacification"). Additionally, Medical Subject Headings (MeSH) terms were used to ensure comprehensive coverage, including "Device-Related Injuries," "Lenses, Intraocular," and "Yttrium Aluminum Garnet”. This systematic review was not prospectively registered in the International Prospective Register of Systematic Reviews (PROSPERO), as registration is neither mandatory nor required for non-interventional reviews without primary data collection; furthermore, no new studies involving human participants were conducted, and only previously published data were analyzed (Figure 1).

We included peer-reviewed publications in English or German that reported experimental studies (in vitro analyses, microscopy, or imaging studies), clinical case series, case reports, reviews, or guideline documents directly related to Nd:YAG-induced IOL damage (for example, pits, craters, cracks, optical degradation, clinical consequences, or preventive strategies). Exclusion criteria were non-peer-reviewed literature, editorials, and publications not available in English or German. In addition, articles that addressed PCO in general without evaluation of Nd:YAG-associated IOL damage, or studies that focused solely on the epidemiology or prevention of PCO without reporting IOL surface alterations, were excluded.

## Review

Nd:YAG laser-induced IOL damage presents with a spectrum of morphologic alterations, varying in depth, shape, and regularity. Superficial pitting appears as shallow, round indentations typically located at or near the posterior optic surface, especially when the laser focus is just anterior to the capsule. More extensive crater formation and material delamination have been reported in cases where high pulse energy (>2 mJ) was used or the focal point was too close to the IOL [17-19].

Hydrophobic acrylic IOLs demonstrate higher susceptibility to deep, irregular pit formation compared to hydrophilic acrylic or polymethyl methacrylate (PMMA) materials [20]. Scanning electron microscopy (SEM) and micro-computed tomography (µCT) have revealed sharp-edged defects with depth penetration exceeding 600 µm in some cases [21]. Raman spectroscopy further identified local chemical degradation within the pit margins, suggesting thermal or mechanical alteration of polymer bonds [22].

Borkenstein et al. investigated pit volume and surface topography across material types and demonstrated in experimental studies that hydrophobic acrylic lenses formed larger and more chaotic crater zones than their hydrophilic counterparts [17, 20-23]. In the case of pinhole IOLs, laser-induced damage to the central carbon ring was more pronounced and had catastrophic results, with a crater expansion involving adjacent structures and disrupting the aperture geometry, a defect described as a "carbon-burst" pattern [23]. Bucur et al. provided clinical confirmation of these findings by documenting a patient case where a 2.7 mJ misaligned Nd:YAG shot led to central distortion of such a pinhole IOL (IC-8) and ultimately required IOL exchange due to functional visual impairment [24].

Optical consequences

Moreover, multiple experimental studies have demonstrated that even minor surface alterations from Nd:YAG laser pulses significantly impair optical performance. Using modulation transfer function (MTF) assessments, Borkenstein et al. observed that centrally placed pits resulted in measurable contrast sensitivity loss, especially under mesopic conditions [25,26]. In high-resolution United States Air Force (USAF) target analysis, pits induced by as few as five laser shots at 1.8 mJ caused detectable image blur and degradation of edge sharpness.

Wavefront aberrometry studies by Rozema et al. highlighted that optical quality loss is closely correlated with the size and location of the defect, with hydrophobic lenses exhibiting more pronounced higher-order aberrations post laser [26, 27]. In simulations of presbyopia-correcting intraocular lenses, YAG-induced pits caused a marked decrease in image contrast (ranging from 52% to 66%) and significant spectral attenuation across wavelengths from 450 nm to 800 nm, highlighting substantial functional impairment under typical viewing conditions. Regarding the authors, in diffractive and multifocal IOLs, even small central defects could lead to overlapping diffraction rings and a notable reduction in near- and intermediate visual function in simulations. Spectral transmittance measurements have also shown that deeper pits scatter a larger portion of the visible spectrum, potentially leading to chromatic distortion and reduced color discrimination in low-light environments. Such optical interference is particularly relevant in patients with small pupils or in nighttime driving situations [25-28].

Clinical relevance

Clinically, Nd:YAG-induced IOL damage may remain asymptomatic in some patients, particularly when defects are small or peripherally located. However, centrally located pits, even if small, have been shown in experimental laboratory studies to negatively impact optical quality, leading to measurable glare, halos, starbursts, and reduced contrast sensitivity [29,30]. Despite these experimental findings, robust clinical evidence demonstrating a direct correlation between such defects and patient-reported symptoms remains scarce. Clinical studies specifically addressing the symptomatic effects of centrally located YAG-induced pits are limited or lacking altogether, and available clinical reports often originate from isolated case reports presented primarily as posters or oral presentations at scientific conferences rather than as full peer-reviewed publications. This gap underscores the need for well-designed clinical studies to clarify the functional consequences of Nd:YAG laser-induced damage, especially in patients with multifocal, toric, or small-aperture lenses, where visual quality depends critically on precise light distribution and focal-zone integrity.

In a retrospective case series, Newland et al. found that approximately 9.3% of the lenses removed due to significant postoperative complications exhibited deep Nd:YAG-induced pits exceeding 300 µm in depth, suggesting that such laser-induced lesions can indeed contribute significantly to visual impairment necessitating IOL explantation [19]. Importantly, also, misdiagnosis of primary IOL opacification as posterior capsule opacification can lead to unnecessary laser treatments according to the authors.

Bucur et al. presented a case in which a pinhole IOL (IC-8) required explantation following a single misaligned YAG pulse that caused severe functional central distortion [24]. Another case report showed that Nd:YAG laser pulses near the pigmented ring of the IC-8 IOL can cause cavitation bubbles, resulting in vacuolation and subsequent lens damage depending on energy levels. To minimize this risk, authors recommended using an Nd:YAG capsulotomy contact lens, allowing a lower laser energy compared to the no-contact technique [25]. Several other cases of IOL opacification mimicking PCO have been reported during oral presentations or posters at conferences, which may lead to unnecessary visual decline after Nd:YAG capsulotomy, though robust, peer-reviewed data are lacking.

Table 1 summarizes the current evidence on Nd:YAG laser-induced IOL damage characteristics according to lens material, detailing reported pit depths, optical consequences, and clinically significant explantation rates derived from peer-reviewed experimental and clinical studies.

**Table 1:** Material-specific characteristics of Nd:YAG laser-induced damage in intraocular lenses: pit depths, optical effects, and clinical relevance IOL: intraocular lens; PMMA: polymethyl methacrylate; Nd:YAG: neodymium-doped:yttrium-aluminum-garnet

IOL Material Type	Reported Pit Depth	Optical Consequences	Clinical Relevance (Explantation Rates)	References
Hydrophobic acrylic	>600 µm	Significant contrast loss, increased glare, higher-order aberrations	Higher susceptibility, severe visual symptoms; explantation up to ~9.3% in severe cases	[20, 21, 25, 17]
Hydrophilic acrylic	<300 µm	Moderate contrast loss, lower aberration increase compared to hydrophobic lenses	Lower clinical severity; rare cases of explantation described	[20, 21, 17]
Small-aperture IOL	Severe (>600 µm)"carbon-burst" damage	Extreme optical degradation, severe visual axis disturbances	High risk of explantation after misfocused pulses (case reported)	[23, 24]
Silicone IOL	Typically 200–400 µm;deep pits >300 µm reported	Moderate scattering, glare, halos, reduced contrast sensitivity	Pit-related explantation rate ~9.3% reported	[19]
PMMA	100–300 µm typically; occasionally >300 µm	Lower optical impact, mild scattering	Rarely explanted solely due to pit formation	[18]

Table 2 provides an overview of reported clinical complications associated with Nd:YAG laser capsulotomy, highlighting their clinical impact, estimated incidence, and relevant references drawn from peer-reviewed literature.

**Table 2:** Clinical complications following Nd:YAG laser capsulotomy: clinical impact, estimated incidence, and relevant literature Nd:YAG: neodymium-doped:yttrium-aluminum-garnet

Complication	Description of Clinical Impact	Estimated Incidence	References
Intraocular lens (IOL) damage (pitting, cracks)	Optical quality loss (glare, halos, reduced contrast); may require lens explantation	Pit formation is common (up to 20%–40%) and clinically significant explantations ~9.3% reported	[17, 20, 21, 23, 24]
Intraocular pressure (IOP) spikes	Acute elevation of IOP immediately after procedure; may be transient or persistent	Common transient occurrence, long-term IOP elevation less frequent	[7, 8, 9]
IOL subluxation/dislocation	Mechanical destabilization of lens position due to shockwave effects	Rare, case-specific events	[6, 7]
Cystoid macular edema (CME)	Inflammatory response leading to macular swelling, transient visual impairment	Infrequent, typically resolves with medical treatment	[3, 9]
Anterior vitreous prolapse	Rupture of the posterior capsule may result in vitreous prolapse	Rare, mostly with high-energy pulses or misaligned laser shots	[9, 17]
Pigment dispersion/inflammation	Pigment release or anterior chamber inflammation post-laser	Relatively common, usually mild and transient	[6, 7, 8]
Retinal detachment	Potential risk due to vitreous disturbance or mechanical stress	Very rare (<1%), mostly in predisposed eyes	[9]
Endophthalmitis	Severe intraocular infection post procedure	Extremely rare (<0.1%), case-reported	[9]

Mechanisms

Damage mechanisms include photodisruptive energy deposition at the IOL interface, generation of plasma and shockwaves, and thermal or mechanical stress propagation through the optic material. In pigmented or carbon-rich IOL zones (e.g., IC-8), localized heating can induce microfractures and delamination [31, 32].

Polymer degradation and chemical bond cleavage within IOL materials have been experimentally demonstrated when Nd:YAG laser energy reaches or exceeds 10 mJ, potentially resulting in structural weakening and release of toxic monomers. Additionally, repeated low-energy pulses, even below the visual damage threshold, can cumulatively induce subtle, progressive surface alterations and material fatigue. Such sub-threshold changes highlight the importance of precise laser focusing and minimal energy application to prevent long-term optical and structural deterioration of intraocular lenses [33].

Discussion

It seems critical to differentiate true PCO from primary IOL opacification prior to performing Nd:YAG treatment. Misinterpretation may lead to unnecessary laser application and worsened visual function, especially in hydrophilic lenses prone to calcification. Calcification of IOLs, especially hydrophilic acrylic IOLs, represents an infrequent yet clinically significant complication following cataract surgery. This phenomenon is characterized by the deposition of calcium phosphate crystals within or on the lens material, resulting in visual disturbances such as reduced contrast sensitivity, glare, and decreased visual acuity. Calcification has been associated with multiple factors, including material composition, packaging processes, surgical technique, and specific ocular or systemic conditions. Management often necessitates lens explantation, making accurate preoperative differentiation from posterior capsule opacification essential to prevent inappropriate treatments such as unnecessary Nd:YAG capsulotomy [34-36]. Moreover, inadvertent or excessively high-energy laser pulses may necessitate additional surgical interventions such as intraocular lens explantation, increasing the chance of patient dissatisfaction and overall healthcare expenditure. This underscores the clinical and economic importance of precise laser application techniques to minimize Nd:YAG-induced complications. Recent developments in simulation-based training for ophthalmologists, such as virtual-reality simulators designed specifically for Nd:YAG laser posterior capsulotomy, have demonstrated significant potential in improving procedural precision and reducing complications among novice surgeons [37]. Furthermore, competency-based educational assessments incorporating structured feedback have been shown to enhance performance consistency and safety during laser procedures, underscoring the value of standardized training methods [38]. Nd:YAG-induced IOL damage is strongly influenced by laser energy, pulse location, and IOL material composition. Experimental studies by Borkenstein et al. have demonstrated that hydrophobic acrylic lenses exhibit greater susceptibility to pit formation, producing deeper and more irregular defects compared to hydrophilic acrylic lenses. Despite their higher sensitivity to laser-induced damage, hydrophobic lenses generally show superior resistance to PCO. These findings underscore the importance of careful laser-energy selection and precise focal-point positioning, particularly when treating patients with hydrophobic IOLs [17, 20-23]. Auffarth et al. demonstrated that IOL lens design and water content significantly influence both glistening formation and PCO risk factors that may also modify susceptibility to Nd:YAG-induced damage. In vitro studies revealed that hydrophobic acrylic IOLs with higher water content exhibited markedly reduced glistenings compared to standard hydrophobic models, while hydrophilic acrylic lenses showed increased propensity for PCO due to lower capsular biocompatibility [39-41]. Auffarth et al. have shown that IOL calcification can occur in hydrophilic acrylic lenses following vitrectomy procedures involving gas tamponade. Post‑vitrectomy IOLs may develop calcium phosphate deposits, leading to significant optical deterioration and increasing the risk of complications during subsequent Nd:YAG capsulotomy [42].

Multiple preventive strategies to minimize Nd:YAG-induced IOL damage has been established. Peripheral laser patterns, such as circular or cruciate patterns, significantly reduce central optical zone damage by redistributing laser impacts away from the visual axis [43]. Posterior offset focusing (focusing the laser 100-200 µm behind the posterior capsule) effectively reduces the risk of unintended contact between the capsule and the IOL surface, further preventing IOL damage [44]. Maintaining the minimal effective laser energy (<1.8 mJ) helps reduce the severity of pit formation and limits structural damage due to laser-induced shockwaves [20-23].

An overview of effective preventive strategies to minimize Nd:YAG laser-induced damage to intraocular lenses is summarized in Table 3.

**Table 3:** Preventive strategies to minimize Nd:YAG laser-induced IOL damage IOL: intraocular lens; Nd:YAG: neodymium-doped:yttrium-aluminum-garnet

Preventive Strategy	Description	Recommended Parameters	Objective/Effect	References
Peripheral laser patterns	Cruciate or circular patterns peripheral to the visual axis	Avoid central laser impacts	Preserve central optical quality	[43]
Posterior offset focusing	Laser focus placed 100–200 µm behind the posterior capsule	100–200 µm posterior	Reduce pit formation on the IOL surface	[44]
Minimal effective laser energy	Use lowest effective pulse energy	<1.8 mJ	Limit pit depth and structural damage	[20–23]
Structured laser alignment aids	Use of specialized contact lenses or markers for precise alignment	Centration guides and fixation points	Improve laser accuracy and reduce misalignment	Expert consensus and ongoing studies (no formal published study available)

Alignment techniques using two fixation points (e.g., anterior segment calibration markers or capsulotomy contact lenses with centration guides) are routinely advocated in expert consensus and ophthalmic laser practice to enhance Nd:YAG laser aiming precision and reduce the likelihood of misdirected pulses, though formal PubMed-indexed studies evaluating their efficacy are currently lacking.

Limitations

We have searched PubMed/MEDLINE database. The findings of this review are predominantly based on experimental and short-term clinical data. Robust long-term follow-up studies are still rare, thus limiting definitive conclusions regarding clinical significance and patient outcomes. A significant gap in current evidence is the lack of patient-reported outcomes, such as those captured by standardized visual function questionnaires (e.g., Visual Functioning 14 (VF-14) and National Eye Institute Visual Function Questionnaire-25 (NEI VFQ-25)). Future clinical studies should therefore systematically integrate such measures to provide comprehensive insight into the patient-centered impact of Nd:YAG laser-induced intraocular lens damage. Such data would enhance our understanding of how subtle optical degradations affect patients' daily visual tasks and overall quality of life.

## Conclusions

Nd:YAG laser capsulotomy remains an effective and widely used treatment for posterior capsule opacification, yet carries the risk of inadvertent IOL damage with potentially significant clinical implications. This systematic review highlights that the extent and nature of IOL damage, ranging from subtle optical disturbances to severe structural degradation, depend significantly on IOL material properties, lens design, and laser technique, including applied energy, pulse placement, and focusing methods. Hydrophobic acrylic lenses, while advantageous in minimizing PCO formation, exhibit greater susceptibility to laser-induced pits and subsequent optical performance degradation. Small-aperture and other premium IOLs require particularly cautious laser approaches due to their heightened sensitivity to optical disturbances. Preventive strategies, including peripheral capsulotomy patterns, posterior offset focusing, minimal effective laser energy application, and optimized alignment techniques, significantly reduce the risk of clinically relevant IOL damage. However, robust clinical data directly correlating laboratory findings with patient-reported visual outcomes remain limited, underscoring the critical need for such controlled clinical studies to validate these experimental insights. Enhanced training programs utilizing simulation and structured feedback mechanisms should be broadly implemented to further mitigate risks associated with Nd:YAG laser capsulotomy, ultimately improving patient safety, clinical outcomes, and overall healthcare efficiency. In summary, we believe careful attention to laser energy settings, precise focusing, and diligent use of preventive strategies during Nd:YAG capsulotomy are crucial to minimizing pit formation on IOL, particularly “premium” IOLs, as even minor defects in these lenses can lead to significant visual disturbances and patient dissatisfaction.
